# The Impact of Social Deprivation on Paranoia, Hallucinations, Mania and Depression: The Role of Discrimination Social Support, Stress and Trust

**DOI:** 10.1371/journal.pone.0105140

**Published:** 2014-08-27

**Authors:** Sophie Wickham, Peter Taylor, Mark Shevlin, Richard P. Bentall

**Affiliations:** 1 Institute of Psychology, Health and Society, University of Liverpool, Liverpool, Merseyside, United Kingdom; 2 School of Psychology, University of Ulster, Magee Campus, Londonderry, Northern Ireland, United Kingdom; The University of Queensland, Australia

## Abstract

The negative implications of living in a socially unequal society are now well documented. However, there is poor understanding of the pathways from specific environmental risk to symptoms. Here we examine the associations between social deprivation, depression, and psychotic symptoms using the 2007 Adult Psychiatric Morbidity Survey, a cross-sectional dataset including 7,353 individuals. In addition we looked at the mediating role of stress, discrimination, trust and lack of social support. We found that the participants' neighbourhood index of multiple deprivation (IMD) significantly predicted psychosis and depression. On inspection of specific psychotic symptoms, IMD predicted paranoia, but not hallucinations or hypomania. Stress and trust partially mediated the relationship between IMD and paranoid ideation. Stress, trust and a lack of social support fully mediated the relationship between IMD and depression. Future research should focus on the role deprivation and social inequalities plays in specific manifestations of psychopathology and investigate mechanisms to explain those associations that occur. Targeting the mediating mechanisms through appropriate psychological intervention may go some way to dampen the negative consequences of living in an unjust society; ameliorating economic injustice may improve population mental health.

## Introduction

### Social deprivation, inequality and psychosis

Since the 1970s, in much of the developed world, the wealthiest have become increasingly wealthy but the wealth of the collective poor has remained unchanged [1]. The negative implications for health and wellbeing of this increasing inequality are now well documented [2]–[5]. Research has identified a wide range of poor physical and psychological outcomes associated with inequality and social deprivation, including obesity [6], cardiovascular disease [7], anxiety [8], depression [9], suicide [10] and also severe mental health diagnoses, including psychosis [11]. The economic consequences of these inequalities are vast. Marmot [2] has estimated that, in the United Kingdom (UK) alone, the healthcare costs associated with social and economic deprivation exceed £5.5 billion per year. However, the mechanisms responsible for these associations are poorly understood. It seems likely that social stressors impacting on emotional regulation play an important role [12]–[15]. Here we examine associations between indices of deprivation, and serious mental illness, investigating associations with specific symptoms in the non-affective psychosis spectrum, and also with depression and mania. In addition we consider the mediating role of stress, discrimination, trust and lack of social support.

Links between economic deprivation and poor psychological functioning are now well established, with associations found for multiple psychopathologies [8], [10], [16], [17]. Economically deprived areas have excessive numbers of diagnosed individuals with depression [18] and also psychosis [19]–[21]. In the case of depression it is estimated that living in deprivation, poverty and feeling financial strain are all significant risk factors [9]. In the case of psychosis, a significant proportion of the variance (8%) in incidence rates is attributed to characteristics associated with neighbourhood level and environmental characteristics [5]. A recent analysis has suggested that a one standard deviation increase in deprivation in England may confer an increased relative risk of psychosis of 1.28 [17].

These associations have often been explained in terms of reverse causality on the grounds that individuals with a deteriorating mental state seek out environments that are typically urban and deprived due to downward social drift (e.g. [22]). However this explanation seems unlikely as research indicates an association before the onset of the illness and especially with deprivation during childhood [23] with one research finding associations at birth [24]. There is the possibility that drift may originate in earlier generations, accounting for these findings. However, a dose-response relationship has been detected between deprivation and psychosis, with those who spend most time in urban and deprived environments being at highest risk [25], [26]. Moreover, research that has controlled for possible genetic confounding suggests that growing up in deprivation puts individuals at a higher risk of a schizophrenia diagnosis, regardless of genetic risk, and perhaps more importantly for intervention strategies, that growing up with high socioeconomic status (SES) is a protective factor for those who are at genetic risk [27].

Recent studies have suggested that inequality (living in circumstances in which there are large variations in economic wellbeing) may confer risk that is additional to the risk of deprivation alone [28], [29]. Kirkbride et al. [17] investigated the association of social inequality with incidence rates of affective and non-affective psychosis. They used the index of multiple deprivation (IMD; a small area statistic made available by the UK Office of National Statistics) and local Gini coefficients calculated by comparing variation in deprivation within each neighbourhood at a lower, nested level of geography, and found that both were associated with non-affective psychosis but not with affective psychosis. More recently, Tsai et al. [30], in a 10-year longitudinal study found that a combination of individual and neighbourhood economic status predicted extremely poor outcomes for psychosis, so that schizophrenia patients with low SES living in deprived neighbourhoods were at 18–22% higher risk of death than those with high SES living in advantaged neighbourhoods. Schizophrenia patients with low SES living in advantaged neighbourhoods also had better outcomes than their counterparts in disadvantaged neighbourhoods.

One limitation of the above research is that it has generally employed broad diagnostic categories as outcome variables. In the case of psychosis, especially, such categories are problematic because they encompass heterogeneous phenomena, leading some to argue that psychopathology is better understood in terms of a small number of dimensions [31] or at the symptom level [32]. Indeed, existing evidence suggests that some environmental factors may confer risk of specific symptoms, for example that childhood sexual abuse confers a specific risk of auditory verbal hallucinations (AVHs) whereas attachment-disrupting events confer a specific risk of paranoia [33], [34]. However, other risk factors, for example, urbanicity [35], appear to confer risk across the broad positive symptom dimension. For example, Oher et al. [35] considered the association between deprivation and psychotic symptoms in people with first episode psychosis. They found that there were higher levels of reality distortion and depressive symptoms in the most densely populated areas, however no clear association was observed for paranoia. In addition, it was associated with hallucinations. In some cases, for example perceived discrimination, the data is inconsistent, with some studies suggesting an association with all positive symptoms [36] and some suggesting a specific association with non-clinical but not clinical paranoia [37], [38]. These kinds of specificities are important because they provide clues about the mechanisms that may give rise to symptoms. In this paper, we consider whether deprivation might be linked to specific psychotic symptoms.

It seems reasonable to hypothesize that paranoid beliefs may be particularly linked to deprivation because psychological research implicates negative social comparison and experiences of victimisation (arguably more likely in deprived and unequal neighbourhoods) in these beliefs [39], [40]. Consistent with this hypothesis, Mirowsky and Ross [41], in a population survey of residents of El Paso (USA) and Juarez (Mexico) found that paranoid beliefs were associated with urban areas in which individuals felt powerless and victimized, and that external locus of control and lack of trust mediated this association. More recently, in a study of a large student sample, Wickham et al. [42] found that recalled relative deprivation in childhood was associated with paranoid beliefs and hallucination-proneness in early adulthood. However, on investigating possible mechanisms to explain this association (beliefs about injustice, trust and social rank) this study found that these variables were significant mediators for paranoid beliefs but not for hallucination-proneness. Finally, experimental studies have demonstrated that walking through a deprived urban neighbourhood can provoke paranoid thoughts in both psychiatric patients [43] and ordinary people [44].

### Underlying mechanisms between inequality and psychosis

Several underlying mechanism have been hypothesised to explain the pathways from environment risks to psychosis. Impaired nutrition [45] and infections [46] have been suggested to play a role, but with varying and unreliable findings [47]–[49]. Given that that stress is implicated in a range of mental illnesses and, in particular, psychosis [15], [50], [51] an alternative possibility is that experiences of deprivation, especially in highly unequal societies such as the UK or the US, are stress-inducing. Concepts such as status anxiety (SA; [52], [53]), social defeat (SD; [54]) and social exclusion (SE; [55]) have all been proposed as potential mechanisms. Although these are conceptually different, a common theme is the perception of being disadvantaged compared to others and hence excluded. The SD hypothesis, in particular, has gone to some lengths to explain the role of not only deprivation but of other risk factors for psychosis such as childhood trauma, urban upbringing, low IQ, migration and illicit drug use [54], [56]. A recent review suggests that the impact of population density, social fragmentation and deprivation on risk of psychosis may not be directly causal, but explained or modified by individual level social stressors (i.e. cannabis use, social adversity, exclusion and discrimination). However the authors of the review note that good research is lacking in this area [26].

In this study, we aimed to assess the association between deprivation as measured by the index of multiple deprivation (IMD) and specific mental health outcomes, using data from the 2007 Adult Psychiatric Morbidity Survey (APMS2007; [57]). For reasons reviewed above, we hypothesised deprivation to be associated with paranoia. In the light of previous research (e.g. [58]), we also predicted deprivation to be associated with depression. Predictions about hallucinations had to be more tentative. On the one hand, in our previous research [42] with student participants we did find a modest association between IMD of neighbourhood during childhood and hallucination-proneness. However, research on the cognitive processes involved in AVHs has implicated impaired source monitoring as a crucial mechanism [59] and there was no reason to predict that this mechanism would be affected by current exposure to deprived urban environments. Hence we anticipated that any association between deprivation and AVHs would be small or non-existent. Given the lack of association between manic symptoms and urbanicity [60], [61], we also did not predict an association between these symptoms and deprivation.

We also tested four potential mediators of the predicted relationships which were available in APMS2007 and which we selected on the basis of previous research. Because discrimination has been implicated in psychosis in some studies [36] and paranoia in others [37], [38] this was included. Trust was included in the light of evidence that it might be specifically implicated in paranoia [41], [43]. Finally, lack of social support and isolation has been implicated in paranoia [62], [63].

Although our main research focus is the positive symptoms of psychosis, because depression has also been linked to poverty [9], [64] and social deprivation [65], [66] we included it in our models. Finally, because mania was also assessed in APMS2007, and because the research linking urbanicity to affective psychosis and bipolar disorder has been inconsistent [17], [67], we also considered it in our analyses.

## Methods

### Ethics statement

The current study used secondary data therefore direct ethical approval was not required. However the original data collection (APMS2007) received ethical approval from the Royal Free Hospital and Medical School Research Ethics Committee (reference number 06/Q0501/71). Written consent was obtained from all participants.

### Sample

The APMS2007 was carried out between October 2006 and December 2007 by the National Centre for Social Research and the University of Leicester. The survey was commissioned by the NHS Information Centre for Health and Social Care and employed a multistage stratified probability sampling design. Individuals aged 16 years and above living in private households were identified for interview in England using postcodes. From 13,171 eligible households, 7,353 individuals completed the first phase (although a second phase involved clinical interviews with a subsample, these data are not used in this study, with the exception that they contributed to the definition of probable psychosis; see below). Researchers administered computer-assisted interviews and self-completion questionnaires using laptops to obtain data on topics including physical health, mental health, service use, religion, social capital, discrimination and sexual abuse. For more information, see McManus et al [57].

### Dependent variables

#### Psychosis

The APMS2007 screened for psychosis experiences during phase one using the Psychosis Screening Questionnaire (PSQ; [68]), and then interviewed a subsample of participants with the Schedule for Clinical Assessment in Neuropsychiatry (SCAN; [69]) in phase two. Probable psychosis and definite psychosis were binary variables derived from phase one and two data.

Probable psychosis was derived by all those who screened positive for psychosis at phase one from the PSQ and during the SCAN assessment at phase two. These were scored as probable psychosis (given a score of 1). In addition, those who did not have a SCAN assessment but met two or more positive psychosis screening criteria on the basis of their questionnaire responses were also assigned a probable psychosis outcome. A score of 0 was given to individuals who only met one positive screening criteria at phase one and who did not have a SCAN assessment, and also to all those who screened as having no psychosis experiences at phase one. The scoring of definite psychosis employed the same strategy as probable psychosis but a different restrained weighting strategy was applied to take account of non-response in phase two (see [57]). This is considered a more conservative estimate of psychotic experiences.

#### Paranoia, AVHs and Mania

The PSQ [68] has 5 sections to identify psychotic-like experiences that may have occurred within the past year: mania/hypomania, thought control, paranoia, strange experiences, and AVHs. Each section has an initial question followed by 1 or 2 follow-up questions to determine severity. For present purposes, paranoia, AVHs and mania/hypomania sections were of interest, and were scored as binary variables, scoring the highest level of severity as 1, and all others as 0. Here, we are using a narrow definition of paranoia, encompassing thoughts of deliberate acts of harm and plotting against an individual as has been used in a previous publication using this dataset [33].

The highest severity of paranoia was assessed using the question, “Have there been times that you felt that a group of people was plotting to cause you serious harm or injury?” The highest severity of auditory-verbal hallucination (AVH) was identified by respondents answering yes to the question: “Did you at any time hear voices saying quite a few words or sentences when there was no one around that might account for it?” The highest severity of mania/hypomania was assessed using the question, “Did people around you think it [being happy, for no reason, for days without a break] was strange or complain about it?”

#### Depression

A binary score for depression (1 = depression, 0 = absent) was derived from the phase one Clinical Interview Scale – Revised (CIS-R, see [70]). This score was derived from the APMS2007 coding script which included many variables from the CIS-R, including depressive ideas and depressive moods, but also fatigue and appetite. The final depression score encompasses mild, moderate and severe depression.

### Mediating Variables

#### Stress

One item from the social functioning section of the APMS2007 asked individuals whether they saw their tasks at home and at work as very stressful. Individuals answered on a 4-point Likert scale from 0 “not at all” to 3 “most of the time”.

#### Social Support

Seven items were used to identify the level of social support each respondent felt that they had from family and friends. Participants were asked to respond “not true” “partly true” or “certainly true” to a series of statements, including whether family and friends did things to make them happy, made them feel loved, could be relied on no matter what, would see that they were taken care of no matter what, accepted them just the way they are, made them feel an important part of their lives, and gave them support and encouragement. Each respondent could have a score between 0 and 14, creating a 15-point scale. In the current analysis, internal consistence is good (Cronbach's α = .88).

#### Discrimination

An eight point scale was derived based on cumulative experiences of discrimination in the last 12 months. This was assessed using eight items from the discrimination section of the APMS2007. Respondents were asked to answer yes (1) or no (0) to identify if they had been unfairly treated in the last 12 months due to skin colour/ethnicity, sex, religious beliefs, age, mental health, other health problems or disability or sexual orientation.

### Independent Variable

#### The Index of Multiple Deprivation

The index of multiple deprivation (IMD) 2004 data were collected for the APMS 2007. This is a derived measure of social and economic deprivation based on seven domains or neighbourhood variables: income; employment; health and disability; education, skills and training; barriers to housing and services; living environment; and crime. Data for the IMD 2004 was collected between 1997 and 2003 and the APMS2007 reported a 5 point scale, where 1 (0.59–8.35) represents the least deprived and 5 (34.21–86.36) represents most deprived.

#### Covariates

Covariates included in the analyses encompassed age (range = 16 to 95 with a mean of 46.35, SD = 18.60). Sex was added as a categorical variable covariate where 48.6% of respondents were male (1 = male, 0 = female). Ethnicity was also added as a categorical variable where 0 equated to white British and 1 equalled other ethnicity. In this dataset 84.6% of respondents were white British. Drug use with the last year was added as a covariate, this was added as a binary variable (1 = yes and 0 = no). In this sample 92% of the total sample reported using any drug in the year leading up to the interview. Highest education level achieved was also entered into the analysis, this included 6 categorical variables from degree (18.6%), teaching, HND and nursing (7.3%), A levels (12.7%), GCSE or equivalent (24.5%), foreign or other (3.9%) and no qualification (30.8%), with 2.3% missing data. Finally AVHs were entered as a covariate in the mediation analysis, this was to address the issue of co-occurrence of symptoms, particularly between paranoia and AVHs) as has been previously identified and used in these large epidemiological datasets (see [33], [34]).

### Statistical Analyses

Descriptive statistics were produced using SPSS 21 and regression and multivariate mediation models were specified and estimated using Mplus6.1. Logistic regressions were carried out to assess the association between the independent variable (IV) IMD and the dependent variables (DV) mental health outcomes (probable psychosis, definite psychosis, depression, paranoia, AVHs and mania/hypomania). This was completed three times. In the first regression IMD was regressed to definitive psychosis, as this required different weighting than for other DVs. The second regression allowed IMD to predict probable psychosis, and the final regression allowed IMD to predict to all other DVs (depression, paranoia, AVHs and mania/hypomania). All regressions used the appropriate weighting for survey design and non-response in order to be representative of English population (see [57]).

**Figure 1:**
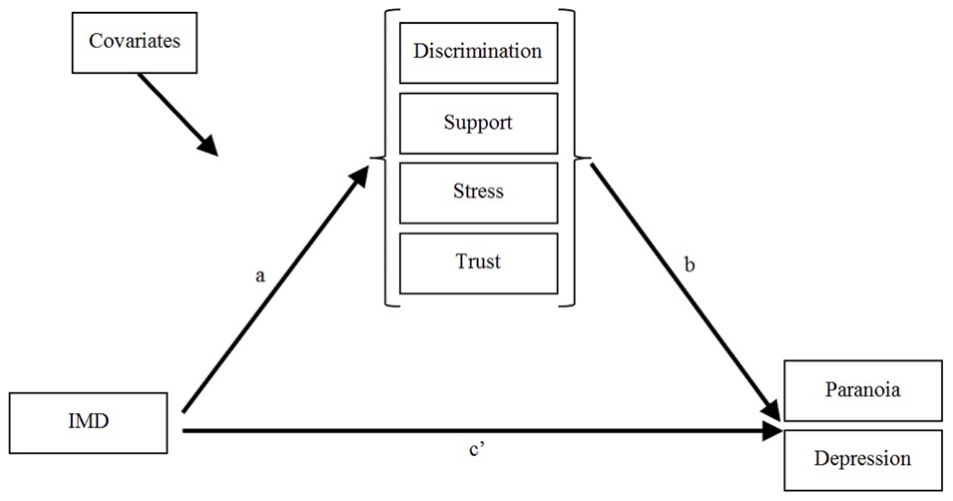
Illustration of the final model (model 3) used in the analysis. Please note that the mediators representing discrimination, support, stress and trust were allowed to covary. Covariates included age, sex, ethnicity and hallucinatory experiences. The mediating variables (discrimination, support, stress and trust) were regressed onto the IV - the index of multiple deprivation (IMD) variable (path a). The DVs (paranoia and depression) were regressed onto the mediating variables (path b) and the DVs were also regressed onto the IV IMD (path c′) simultaneously. The effects from IMD to the mediating variables were linear regression estimates and the effects from the mediators to the symptoms were logistic estimates reported as odds ratios.

Mediation was investigated using multivariate binary logistic regression models estimated in Mplus 6.1 [71] using robust maximum likelihood estimation. In in an initial model, direct paths between the IMD and the symptoms associated with IMD were computed. Model 2 added the direct paths from covariates to symptoms including age, sex, ethnicity and AVHs. AVHs were added as a covariate due to its shared variance with both paranoia and depression [33], [34]. All parameters were estimated simultaneously so that the differences in the estimates for the IMD variable between model 1 and model 2 showed the effects of controlling for comorbidity between symptoms and other potential confounders. In model 3, discrimination, stress, lack of support and trust variables were introduced in the model as mediators between IMD and symptom outcome variables, and the mediating variables were allowed to covary. The effects from IMD to the mediating variables were linear regression estimates and the effects from the mediators to the symptoms were logistic estimates reported as odds ratios (see Figure 1).

The adequacy of each model was assessed by examining three information theory based fit statistics: the Akaike Information Criterion (AIC; [72]), the Bayesian Information Criterion (BIC; [73]), and the sample size adjusted Bayesian Information Criterion (ssaBIC; [74]) with lower values indicating better model fit. These fit statistics balance model fit with parsimony in order to determine the optimum model. In addition, chi-square difference tests were used to determine the best fitting model.

## Results

Simple bivariate associations between IMD and mental health outcomes are summarized in Table 1. The general trend suggests that as deprivation increases the presence of mental health difficulties also increases. This is reflected in the logistic regression analyses (Table 2), when controlling for age, sex, ethnicity, drug use in the last year and education level achieved, IMD predicts definite and probable psychosis (*ß* = 34, *p*<.01; *ß* = .26, *p*<.01 respectively), as well as depression (*ß* = .16, *p*<.001). However when we investigated specific symptoms experienced within psychosis (paranoia, AVHs and mania/hypomania) direct associations were found for paranoia (*ß* = .15, *p*<.01) and not for either AVHs or mania/hypomania.

**Table 1:** Simple bivariate associations, showing percentages (%) and actual numbers (n) between the index of multiple deprivation (IMD) and mental health outcomes (psychosis, depression, paranoia, auditory-verbal hallucinations (AVHs) and mania).

	Level of deprivation											
Diagnoses/Symptom	Least deprived						Most deprived					
	0.59>8.35		8.35>13.72		13.72>21.16		21.16>34.21		34.21>86.36		Total	
	%	*(n)*	%	*(n)*	%	*(n)*	%	*(n)*	%	*(n)*	%	*(n)*
Definite Psychosis	4.3	*(1)*	8.7	*(2)*	17.4	*(4)*	17.4	*(4)*	52.2	*(12)*	100	*(23)*
Probable Psychosis	5.0	*(2)*	10.0	*(4)*	22.5	*(9)*	22.5	*(9)*	40.0	*(16)*	100	*(40)*
Depression	12.5	*(32)*	11.0	*(28)*	14.9	*(38)*	27.5	*(70)*	34.1	*(87)*	100	*(255)*
Paranoia	6.4	*(8)*	9.6	*(12)*	24.8	*(31)*	28.0	*(35)*	31.2	*(39)*	100	*(125)*
AVHs	14.7	*(10)*	10.3	*(7)*	20.6	*(14)*	26.5	*(18)*	27.9	*(19)*	100	*(68)*
Hypomania	9.1	*(4)*	15.9	*(7)*	22.7	*(10)*	22.7	*(10)*	29.5	*(13)*	100	*(44)*

**Table 2:** Summary of logistic regression analysis for variables predicting mental health outcomes, controlling for background variables (age, sex, ethnicity, education and drug use).

	Unstandardised		Standardised		
Index of Multiple Deprivation to:	ß	SE	ß	SE	OR (95% CI)
Definite Psychosis	.57*	.20	.34*	.11	1.8* (1.2–2.6)
Probable Psychosis	.39*	.14	.26*	.08	1.5** (1.1–1.9)
Depression	.21**	.06	.16**	.04	1.2** (1.1–1.4)
Paranoia	.22*	.08	.15*	.05	1.2** (1.1–1.5)
AVHs	.12	.11	.09	.08	1.1 (0.9–1.4)
Mania	.09	.14	.06	.10	1.1 (0.8–1.4)

Note:

**p<.001,

*p<.01 Definitive Psychosis was weighted using specific weight designed for this variable; all other variables were weighted using phase one data weights.

The odds ratios (ORs) from the multivariate binary logistic regression analysis are presented in Table 3. Model 1 estimated the direct effect of IMD on paranoia and depression. For this model the likelihood ratio chi-square was statistically significant (*X^2^* = 54.94, df = 12, *p*<.01). The addition of the covariates (model 2; change in *X^2^*(Δ*X^2^*) = 186.61, change in df (Δdf) = 12, *p*<.01) and the mediator variables (model 3; Δ*X^2^* = 2583.82, Δdf = 38, *p*<.01) made significant improvements to the overall model (Table 3).

**Table 3:** Logistic regression odds ratios of symptoms by IMD and fit indices for the mediation model.

Variable		Model 1 OR (95% CI)	Model 2 OR (95% CI)	Model 3 OR (95% CI)
Paranoia	IMD	1.44** (1.25–1.66)	1.26** (1.07–1.47)	1.19* (1.02–1.40)
	Age	-	0.97** (0.96–0.99)	0.99* (0.97–1.00)
	Sex	-	1.35 (0.88–2.09)	1.36 (0.87–2.11)
	Ethnicity	**-**	2.47** (1.48–4.13)	2.24** (1.32–3.78)
	Education	**-**	0.93 (0.83–1.04)	0.97 (1.14–3.63)
	Drug use	**-**	2.35** (1.34–4.11)	2.03 (1.14–3.63)
	AVHs	**-**	22.78** (10.21–50.85)	12.08** (5.07–28.81)
	Discrimination	**-**	-	1.47** (1.07–2.03)
	Stress	**-**	-	1.41** (1.13–1.75)
	Trust	**-**	-	1.45** (1.19 - 1.76)
	Support	**-**	-	1.05 (0.98–1.14)
Depression	IMD	1.29** (1.16–1.44)	1.23** (1.09–1.39)	1.11 (0.98–1.26)
	Age	-	0.99** (0.99–1.00)	1.01 (1.00–1.02)
	Sex	-	0.66** (0.49–0.90)	0.63 (0.45–0.87)
	Ethnicity	**-**	0.85 (0.53–1.38)	0.62 (0.37–1.05)
	Education	**-**	0.88** (0.81–0.95)	0.91* (0.84–0.99)
	Drug use	**-**	2.02 (1.29–3.18)	1.54 (0.92–2.57)
	AVHs	**-**	7.18** (3.43–15.02)	2.15 (0.92–5.01)
	Discrimination	**-**	-	1.96** (1.56–2.46)
	Stress	**-**	-	2.30** (1.95–2.70)
	Trust	**-**	-	1.34** (1.18–1.53)
	Support	**-**	-	1.10** (1.04–1.16)
Loglikelihood		-39894	-39801	-38509
# Free Parameters		12	24	62
AIC		79813	79650	77142
BIC		79895	79815	77568
ssaBIC		79857	79739	77371

Note:

**p<.01,

*p<.05.

IMD (Index of multiple deprivation), AVHs (auditory-verbal hallucinations), AIC = Akaike information criterion, BIC = Bayesian information criterion, SSABIC = sample size adjusted BIC.

In model 1, the IMD variable significantly predicted each symptom (paranoia and depression). The effect was highest for paranoia, with an OR of 1.44 (95% CI [1.25, 1.66]). The OR for depression was 1.29 (95% CI [1.16, 1.44]). The introduction of the covariate variables in model 2 resulted in a slight decrease in the ORs, however remained statistically significant (paranoia OR = 1.26, 95% CI [1.07, 1.47]; depression OR 1.23, 95% CI [1.09, 1.39]).

In model 3, the IMD variables significantly predicted the mediating variables stress, (standardised *B* = 0.03, *SE* = 0.01, *p* = .01) lack of trust (*B* = 0.23, *SE* = 0.01, *p*<.001), lack of social support (*B* = 0.04, *SE* = 0.01, *p*<.001), and discrimination (*B* = 0.03, *SE* = 0.01, *p* = .02). Each of the potential mediators significantly predicted the symptom measures with the exception of the path from lack of support to paranoia, which was non-significant. The largest effect for paranoia was discrimination (OR = 1.47, 95% CI [1.07, 2.03]) and the smallest effect was stress (OR = 1.41, 95% CI [1.13, 1.75]). With the DV depression, the largest effect was between stress and depression (OR = 2.30, 95% CI [1.95, 2.70]). The smallest effect was between lack of social support and depression (OR = 1.10, 95% CI [1.04, 1.16]). Model 3 indicates that *partial mediation* occurred between IMD and paranoia through discrimination, stress and lack of trust. This is due to the fact that c′ (the relationship between IMD and paranoia) remained significant, but reduced between models 2 and 3 (from OR = 1.26, *p*<.01, to OR = 1.19, *p*<.05). However, in the case of depression, *full mediatio*n occurred as model 3 shows that c′ became non-significant when the mediators were included.

## Discussion

Consistent with our first hypothesis neighbourhood deprivation significantly predicted psychosis and depression. When focussing on specific symptoms, again as we hypothesised, IMD was associated with both paranoia and depression, but not with AVHs or mania. In our multivariate mediation model, when controlling for comorbidity between the symptoms and other background variables (age, sex, ethnicity, drug use and education level), IMD was associated with all four mediators; lack of trust, social support and stress and discrimination. Examination of the final model (model 3) indicated that the ORs for the direct effects between deprivation and symptoms reduced once the mediators were entered into the model. In the case of depression the OR became statistically insignificant, indicating full mediation between IMD and depression through discrimination, trust, lack of support and stress [75]. In the case of paranoia, the OR reduced in magnitude but remained statistically significant therefore indicating partial mediation through discrimination, trust, and stress. Overall, this study is generally consistent with previous findings of an association between social and economic deprivation and psychosis [11], [17], [27]–[29] but our analyses add to previous studies by indicating that this association relates to specific symptoms and can be at least partially explained by several potential underlying mechanisms.

The absence of significant affects for both hallucinations and mania are as worthy of note as the positive findings. As discussed earlier, impairment in the ability to discriminate between self-generated thoughts and external verbal stimuli, a process known as source monitoring, is a crucial mechanism in vulnerability to auditory hallucinations [59], [76]. Although it is well known that childhood trauma is associated with hallucinations in adulthood [33], and although we had previously found an association between childhood IMD and hallucination-proneness in early adulthood, there was no reason to believe that source monitoring would be affected by current exposure to urban environments as measured in this study.

Very little is known about environmental risk factors for mania, but there is evidence that manic episodes can be triggered by goal achievement life events [77] which are, if anything, less likely to be experienced in deprived versus affluent areas. Previous research has failed to find any association between bipolar disorder and urban environments [67] and our findings appear to confirm this observation.

Two findings from covariates included in our analyses are also worth considering. Female sex was associated with an increased risk of depression, as shown in many previous studies [78]. This observation therefore testifies to the validity of our approach. Perhaps more intriguingly, belonging to an ethnic minority group was strongly associated with paranoia but not depression. It has been well-demonstrated that ethnic minority status is a risk factor for psychosis [79] but we are not aware of attempts to tease out which positive symptoms of psychosis are most sensitive to this effect. Because this observation emerged in models which also include trust, stress, social support and discrimination, the implication is that this increased risk from ethnicity cannot be explained by these variables. Further research is required with other datasets to determine whether a specific link between ethnic status and paranoia can be replicated, and what mechanisms might be involved.

A number of limitations to the study need to be considered. Firstly, the PSQ used in the APMS2007 dataset concerns experiences in the last year, rather than across the lifetime. It is possible that people classified as not experiencing paranoia or AVHs in our analyses in fact had these experiences in the more distant past. However, it is worth noting that focusing on recent experiences of psychosis as opposed to lifetime experiences will have most likely led us to underestimate the association between deprivation and symptoms. Second, the measure of social deprivation used in the APMS2007 dataset, IMD scores was divided into quintiles, producing an ordinal scale; due to data protection restrictions raw IMD data was unavailable to us. This loss of information may have attenuated some relationships. Moreover, the measure pertained to current living circumstances, rather than those of, say, childhood or the time of onset of psychotic experiences. Hence we hypothesize that the findings reflect the provocation of psychotic symptoms by the current environment (as in the case of the experimental studies of the effects of the urban environment on paranoia, [43], [44]), rather than developmental pathways to psychotic symptoms. Third, the reliability of our measure of stress may be compromised as it used a single item relating to work stress. Arguably, the findings related to stress need to be assessed using an established measure of stress to incorporate other forms of stress (e.g. emotional and home stressors).

Fourth, and perhaps most importantly, the present study is cross-sectional and correlational, and direction of causality cannot be tested using the statistical models we have employed. Therefore, we cannot exclude the possibility that symptoms may affect people's judgments about discrimination, trust, stress and social support or, indeed, that, when answering questions about paranoia and depression, feelings of discrimination, mistrust, stress and the experience of poor social support influenced their answers. Indeed, it could be argued that there is conceptual overlap between mistrust and paranoia and that, therefore, a close association between the two was inevitable. (We think this argument is harder to make about the other mediators.)

With respect to our meditational models, there is no certainty that our mediators were temporally consequent to exposure to a deprived urban environment or that they preceded the onset of symptoms. However, it seems unlikely that people suffering specifically from paranoia and depression (as opposed to AVHs and mania/hypomania) would seek out deprived neighbourhoods to live in. Moreover, our findings are not only consistent with our meditational hypotheses, but also with our previous findings on the relationship between perceived social disadvantage and paranoia [42], with current understandings of the psychological mechanisms underlying paranoid beliefs [39], [40], and, as already noted, with experimental studies which show that short-term exposure to deprived environments tends to provoke paranoid thoughts [43], [44]. A further caveat is that epidemiological findings, particularly involving mediation analysis [80], are vulnerable to unmeasured covariates. This is a problem in most areas of research that can only be addressed by considering other potential covariates in future studies.

It might be argued that our results offer some support to the SD hypothesis and related concepts [54], [56], suggesting that living at the bottom end of an unequal society breeds stress and a lack of trust and support a constellation of experiences which might be described as socially defeating. However, one criticism of the SD model is that it is fairly non-specific in terms of what characterises social defeat, which symptoms are most affected and likely mediating mechanisms.

To our knowledge this is the first study to test for specific symptom relationships (paranoia and depression) with deprivation, as opposed to the broadly defined diagnostic categories which have been identified as particularly problematic when researching social determinants of psychopathological phenomena [81]. It will be useful to replicate these findings in other samples to further assess the specific associations identified here. Longitudinal designs would also help clarify the direction of causality. If supported by future research the present findings have important social implications.

Although the effects found in paranoid and depressive symptoms in this study might be described as modest, at the population level the impact of deprivation on mental health is potentially large. If we consider the UK as an example, the inevitable outcome of experiences of deprivation indicates that more people are being pushed towards mental illness [82]. In much of the developed world, income inequalities have increased since the 1970s with current disparities at an all-time high [2]. It may be possible that efforts to reverse this trend, for example through progressive taxation or through enhanced benefits to poor families will, in the long-term, positively impact on public mental health but devising such policies, and gaining popular support for their implementation may prove to be a very considerable challenge for all concerned, particularly as individuals tend to justify the status quo in more unequal societies [83], [84]. It may be more viable to tackle the social stressors that inequality produces through psychological treatments (e.g. cognitive-behaviour therapy (CBT) and interpersonal psychotherapy) which already aim to address self-esteem and are thought to be effective in depression [85], and in the case of CBT at least, effective for psychosis (CBTp [86]). It is possible that these might be enhanced by specifically targeting the mediating mechanisms outlined in the current study, namely support, trust and stress. At a societal level, our findings suggest that public mental health might be promoted by either addressing inequalities, ameliorating the social stressors that arising from inequalities, or both.
